# Reliability Analysis of the Proactive Transmission of Replicated Frames Mechanism over Time-Sensitive Networking

**DOI:** 10.3390/s21248427

**Published:** 2021-12-17

**Authors:** Inés Álvarez, Manuel Barranco, Julián Proenza

**Affiliations:** Department of Mathematics and Informatics, University of the Balearic Islands (UIB), 07122 Palma, Spain; manuel.barranco@uib.es (M.B.); julian.proenza@uib.es (J.P.)

**Keywords:** reliability analysis, time redundancy, Proactive Transmission of Replicated Frames, Time-Sensitive Networking

## Abstract

The Time-Sensitive Networking (TSN) Task Group has standardised different mechanisms to provide Ethernet with hard real-time guarantees and reliability in layer 2 of the network architecture. Specifically, TSN proposes using space redundancy to increase the reliability of Ethernet networks, but using space redundancy to tolerate temporary faults is not a cost-effective solution. For this reason, we propose to use time redundancy to tolerate temporary faults in the links of TSN-based networks. Specifically, in previous works we proposed the Proactive Transmission of Replicated Frames (PTRF) mechanism to tolerate temporary faults in the links. Now, in this work we present a series of models of TSN and PTRF developed using PRISM, a probabilistic model checker that can be used to evaluate the reliability of systems. After that, we carry out a parametric sensitivity analysis of the reliability achievable by TSN and PTRF and we show that we can increase the reliability of TSN-based networks using PTRF to tolerate temporary faults in the links of TSN networks. This is the first work that presents a quantitative analysis of the reliability of TSN networks.

## 1. Introduction

The Time-Sensitive Networking (TSN) Task Group (TG) has been working on providing Ethernet with hard Real-Time (RT) guarantees and Fault-Tolerance (FT) mechanisms in layer 2 of the network architecture [1]. To that end, the TG has developed a series of technical standards that can be combined to design the most adequate network architecture for virtually any application. These new features together with the well-known advantages of Ethernet, e.g., low cost, high bandwidth, IP compatibility, have drawn the attention of industry and academia. In fact, TSN standards could become the network technology of the future distributed industrial systems, replacing the proprietary solutions that are currently being used [2].

Some of the areas targeted by the TSN TG are automotive [3], industrial automation [4] and aerospace [5]. Many of the novel applications being developed in these areas are subject to tight timing and reliability constraints. On the one hand, some of these applications interact with the environment in which they operate and, thereby, they need to provide adequate services within the time dictated by the dynamics of the system, i.e., they must operate in real time. On the other hand, some of the applications are critical, as their failure could have catastrophic consequences. In these cases, the applications must provide their services correctly during their mission time with a high probability, i.e., they must be reliable. Examples of these applications are autonomous driving or space exploration missions. Furthermore, when these applications are executed in a distributed manner their communication network must also provide RT guarantees and reliability. Specifically, the communication network must ensure that all frames are delivered within a bounded time (real-time) with a high probability (reliability).

In order to increase the reliability achievable by Ethernet networks, the TSN TG has completed the standardisation of two fault tolerance mechanisms. The first mechanism enables the use of space redundancy [6,7], i.e., it allows for an end-system to send each frame through several communication paths in parallel. The second mechanism implements error containment, which allows detecting incorrect frames and reducing the negative impact that they may have on the network [8].

Nevertheless, the TSN TG has not proposed any time redundancy mechanisms in layer 2 of the network architecture to tolerate temporary faults in the links. Even though temporary faults can be tolerated using space redundancy, it is not always the most adequate solution nor the classical approach for tolerating temporary faults. Since space redundancy requires the addition of hardware to the network, using it to tolerate temporary faults may not be cost-effective and it increases the size, weight, cabling routing, installation effort and energy consumption of the network subsystem.

Therefore, in previous works we proposed to use time redundancy to tolerate temporary faults in the links, as we think it is a more suitable solution, specifically for systems with cost, size, weight, or energy restrictions. Specifically, we have designed the Proactive Transmission of Replicated Frames (PTRF) mechanism to provide time redundancy to TSN-based networks. PTRF consists in transmitting several copies (replicas) of each frame in a preventive manner, to increase the chances of each frame reaching its destination. We have designed three different approaches to PTRF. We presented the first two, namely A and B, in [9]; whereas we proposed the third one, *C*, in [10]. Furthermore, we published a detailed description of all the PTRF mechanisms in [11].

Despite the interest in providing TSN networks with fault-tolerance mechanisms, to the best of the authors’ knowledge, there are no previous works that quantify the reliability that these mechanisms can yield. In fact, most of the works that treat space redundancy in TSN focus on evaluating the impact on the performance, bandwidth or schedulability of the network. Furthermore, even the works that do focus on dependability aspects do not provide a quantitative measure for the reliability. Instead, they analyse it in a qualitative manner or focus on specific gains in the fault tolerance capabilities, e.g., counting the number of simultaneous faults that can be tolerated.

We model and quantitatively compare the reliability benefits that can be obtained in TSN networks when using time redundancy. More specifically, we compare the reliability when using TSN with no space redundancy, when using approach A of PTRF and when using approach B. To that aim, we carry out a parametric sensitivity analysis that allows us to quantify how several dependability-related aspects of PTRF affect the reliability in the presence of temporary faults.

The remainder of the article is structured as follows. Section 2 presents an overview of works related to this one and Section 3 presents a description of the TSN standards that are relevant to understanding this work. Section 4 provides an overview of the PTRF mechanism, focusing on those aspects that are key to carrying out our reliability analysis. Section 5 thoroughly describes the most relevant aspects of the PRISM models developed during this work, including the reliability metric, the fault model, the modelling assumptions, the modelling strategy and the model testing; Section 6 presents the parameters that can be tuned in our models and the values we have used for our analysis and Section 7 show the results of our sensitivity analysis of the reliability achievable by TSN and PTRF. Finally, Section 8 summarises the article and highlights the most important conclusions reached with this work.

## 2. Related Work

At the moment of writing this article, we have published four works related to the PTRF mechanism. In [9] we present a first overview of the mechanism, we propose two of the approaches and we compare them using a preliminary probability analysis to calculate the number of replicas required by each approach to reach the same reliability. We base our analysis on the Bit Error Rate (BER) of the links and we assume that the occurrence of bit errors follows a Poisson distribution. Even though this first analysis allows us to see which one of the two approaches can provide the highest reliability using a specific number of replicas, it does not allow to quantify the reliability that each approach can yield under different operation conditions.

In [12] we present a proposal to integrate PTRF with the IEEE Std 802.1CB standard for Frame Replication and Elimination for Reliability (FRER) that supports space redundancy in TSN [6,7], while in [10] we present the third approach of the PTRF mechanism, we build a simulation model for each approach and we use exhaustive fault injection to compare them in terms of the number of fault scenarios that each can tolerate. Furthermore, we present a mathematical analysis to calculate the expected fault scenarios that each approach can tolerate.

Finally, in [11] we present a detailed description of the PTRF mechanism, its operation and its implementation in a real TSN bridge. We also present a quantitative evaluation of PTRF, where we measure its impact on the end-to-end delay, jitter and bandwidth consumption; as well as a comparison with a standard TSN network.

We must note that in the particular case of data networks, retransmissions are normally based on ARQ techniques [13], which rely on ACK/NACK messages and/or timeouts to trigger the retransmission of lost frames. However ACKs are not well-suited for RT highly reliable industrial networks. First, due to the random nature of temporary faults, ARQ solutions are non-deterministic in terms of end-to-end delay and bandwidth consumption. Second, the end-to-end delay of a frame significantly increases when, and only when, retransmissions are required, which leads to a high jitter in the communications. Third, ARQ solutions introduce additional scenarios involving faults, which are harder to tolerate [14] as temporary faults can also affect ACK/NACKs. Finally, when the network conveys TT traffic, the schedule must cope with the worst-case scenario involving temporary faults. Since ACK/NACKs have to be scheduled in addition to frame retransmissions, ARQ solutions further worsen the utilisation efficiency of the bandwidth.

So far we have only talked about space redundancy, proactive transmission of replicas and backward error recovery to achieve fault tolerance. Nonetheless, we must note that there are other fault tolerance techniques, such as error correction codes. Error correction codes have been extensively used in wireless networks with really high noise, but they are rarely used in wired ones [15]. This is because when the noise is as low as in wired networks, the time required to produce the correction codes and to decode and rebuild frames is considerably higher than the time required for simple retransmissions.

Furthermore, in some cases correction codes transmit frames in groups to improve channel efficiency. Nevertheless, the size of the group significantly impacts the time response. This is because frames cannot be transmitted immediately after their creation, but they need to be buffered and transmitted when the whole group is ready. Another common technique to improve the efficiency of correction codes consists in transmitting ACK messages, which, as we have already discussed, introduces a high jitter in the communication. For these reasons, error correction codes are not a suitable solution to tolerate faults in wired networks with hard real-time requirements.

We can find in the literature other works related to the study of TSN and its fault tolerance mechanisms. In [16] the authors discuss in a qualitative manner the challenges and limitations of FRER. In [17] the authors study the capability of tolerating the faults caused by single event upsets (a specific type of temporary fault) using a duplicated communication path, showing that an adequate schedulability analysis is required to tolerate all faults even with redundant paths. Finally, in [18] the authors present a simulation model for redundancy management and validate the design of the redundancy mechanism using fault injection. Nevertheless, to the best of the authors’ knowledge there are no works devoted to evaluating the reliability of TSN-based networks in a quantitative manner.

In this article we present three formal models based on the PRISM probabilistic model checker. PRISM has extensively been used to evaluate the reliability of systems, protocols and networks. Some examples of this are [19], where the authors use PRISM to model and evaluate the reliability of a group membership protocol for dual-scheduled time division multiple access networks; or [20], where the authors study the reliability achievable when using fault tolerance mechanisms in an architecture specifically designed for highly reliable adaptive distributed embedded systems.

To the best of the authors’ knowledge, this is the first article that presents a formal model based on the PRISM probabilistic model checker and a parametric sensitivity analysis to evaluate the reliability achievable by standard TSN and the PTRF mechanism in the presence of temporary faults.

## 3. Overview of TSN Relevant Standards

In any distributed system the devices must exchange messages through the network in order to share information. In embedded systems, the devices usually need to exchange the same information periodically, e.g., the value of a temperature sensor every hour. This is what we call the editions of a message or message editions. Before transmission, each edition is embedded with control information in a frame. A frame is a chain of bits that is transmitted through the physical layer.

The TSN TG has already completed the standardisation of many mechanisms to enforce timeliness in the transmission of frames, such as global clock synchronisation [21] or the Time-Aware Shaper (TAS) for time-triggered traffic [22]. Furthermore, the TG has also completed the standardisation of two mechanisms to increase the reliability of the network, one of them for supporting space redundancy [6,7] and the other one for providing error containment to reduce the negative impact that incorrect frames may have on the network [8]. All these mechanisms operate in layer 2 of the network architecture.

**Figure 1:**
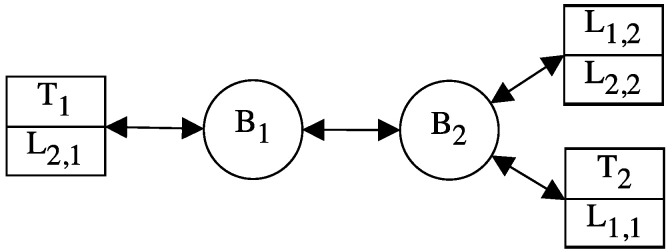
Example of a TSN-based network with three end-systems and two bridges. Two end-systems act as talker and listener and the third one acts as listener of two streams.

In TSN-based networks the communication is carried out through streams. A stream is a logical communication path that connects a transmitter with its intended receivers, to exchange the editions of a message. The different editions of a message usually have the same characteristics, i.e., all the editions have the same size and deadline. Furthermore, in TSN the streams follow a publisher–subscriber model, where the transmitter announces its intention to send a message through a stream and the receivers subscribe to the stream to receive such message. In TSN, publishers are called talkers and subscribers are called listeners. Each end-system can be talker and listener of different streams simultaneously, as depicted in Figure 1, where we can see a TSN network formed by three end-systems and two bridges. From this moment on, when we talk about concepts applicable to both end-systems and bridges, we will use the term network devices.

**Figure 2:**
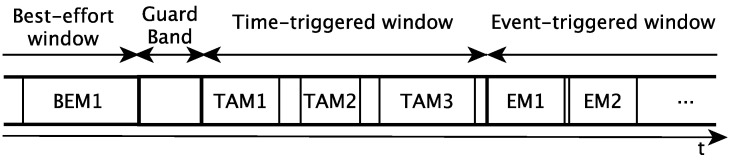
Example of a communication cycle in an output port of a device. The communication cycle is divided into a time-triggered, an event-triggered and a best-effort window.

In order to provide hard real-time guarantees to the communications and to support different traffic classes, TSN relies on TAS. TAS divides the communication time of each network device in cycles, which in turn are divided into windows. Each window is devoted to the exchange traffic of a specific class. Figure 2 depicts an example of communication cycle divided into a time-triggered, an event-triggered and a best-effort window, where TAMi refers to time-triggered message *i*, EMi refers to event-triggered message *i* and BEMi refers to best effort message *i*. Furthermore, the figure depicts a guard band, a time reserved to prevent best effort traffic from interfering with time-triggered traffic.

Since each output port has its communication cycle, to ensure that frames traverse the network in a timely manner, the cycles of all bridges must be coordinated and synchronised. For this purpose, the IEEE Std 802.1Qch [23] proposes the Cyclic Queuing and Forwarding (CQF) mechanism to provide real-time guarantees for the frames transmitted throughout the network. This mechanism roughly consists in assigning a transmission window to each hop (either from one end-system to a bridge, from one bridge to an end-system or between two bridges), in such a way that each frame is forwarded in consecutive windows.

Let us illustrate the operation of CQF with an example. Let us assume that we have the network depicted in Figure 1. Let us also assume that talker of Stream 1, $T_{1}$, wants to communicate with listeners 1 and 2 of Stream 1, $L_{1,1}$ and $L_{1,2}$, using the CQF mechanism. $T_{1}$ transmits frame 1 through the stream 1, $f_{1,1}$, in the communication window $w_{i}$, and $f_{1,1}$ reaches bridge $B_{1}$ within the same window. In turn, $B_{1}$ forwards frame $f_{1,1}$ during window $w_{i+1}$, and the frame reaches bridge $B_{2}$ within that same window again. Finally, bridge $B_{2}$ forwards frame $f_{1,1}$ during window $w_{i+2}$, and the frame reaches listeners $L_{1,1}$ and $L_{1,2}$ within window $w_{i+2}$. Thus, the maximum end-to-end delay of a frame corresponds to the window size × the number of hops, i.e., it takes three windows for frame $f_{1,1}$ to be transmitted.

When using CQF, we can define the period of any given stream as a multiple of communication windows, as long as all the windows in the path have the same duration. Thus, we can define the period *T* of a stream as T=n×w, where *T* is the period, *n* is the number of windows and *w* is the duration of a window. When using PTRF to replicate frames in the time domain, we must take into account that all the copies of a frame (replicas) must fit in the same window when defining *w*, as we explain in Section 6.

## 4. Overview of the Proactive Transmission of Replicated Frames (PTRF) Mechanism

As we have already mentioned, PTRF is a time redundancy mechanism based on the proactive replication of frames. This technique aims at increasing the probability of at least one replica reaching the destination, i.e., increasing the probability that all message editions of a stream reach their destination. If more than one replica reaches the destination successfully, the receiver must detect and eliminate the surplus replicas to deliver only one frame to the application.

Even though there are other time redundancy techniques, proactive replication is deterministic in the time and bandwidth consumption and it allows scheduling time-triggered traffic using the techniques that are already in place in TSN systems. Furthermore, it can be easily integrated with other fault-tolerance mechanisms, as we showed in [12]. Please see [11] for a detailed discussion of all the advantages of proactive replication.

In this section, we describe the fault types and failure modes assumed in the design of the PTRF mechanism and we describe the most relevant aspects of PTRF for this work. For a thorough description of the mechanism the reader can refer to [11].

### 4.1. Fault and Failure Models for the Design of PTRF

In order to properly design a fault-tolerant system, first it is necessary to define its fault model and its failure model. The fault model includes the fault types that are assumed that may happen and, thus, the ones for which the system’s fault-tolerance mechanisms are conceived to. On the other hand, the failure model includes the failure modes the system and its components are supposed to exhibit when suffering the faults included in the fault model. The failure mode of a system or a component refers to the incorrect behaviour that it may exhibit when it suffers from a fault.

Normally, a given fault-tolerance mechanism is not designed to deal with all the types of faults and failure modes of a system. Instead, a given fault-tolerance mechanism is usually devoted to addressing a subset of faults and failure modes. Thus, the mechanism’s fault model and failure model have to be defined accordingly.

The fault type addressed by PTRF can be classified as temporary non-malicious operational hardware faults [24] that affect links, i.e., faults that last for a finite amount of time, that are not caused in a voluntary manner and that affect the hardware components of the system. Some examples of this type of fault are those caused by electromagnetic interference or temperature variations. These are the type of faults most commonly considered when designing communication subsystems.

As concerns the failure model of PTRF, note that PTRF is designed to tolerate temporary faults in the links. Thus, its failure model is constituted by the failure modes of these components. Concretely, we assume that links only exhibit omission failures i.e., we assume that whenever a fault in a link affects a frame, if the frame becomes erroneous then it is eliminated by the communication port upon reception, manifesting as a frame omission. This assumption is reasonable as Ethernet frames count with a Cyclic Redundancy Check (CRC) code, which allows detecting virtually all the errors in a frame [25].

We next describe the operation of the PTRF mechanism, the three different approaches and the most relevant aspects of our design.

### 4.2. PTRF Operation

**Figure 3:**
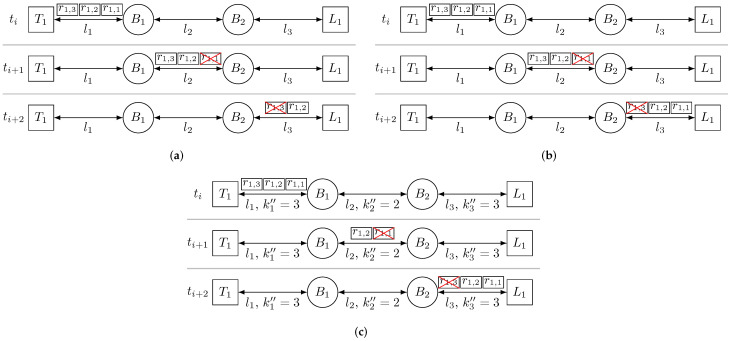
Behaviour of the three approaches of PTRF in the presence of temporary faults in the links. Reproduced as in [11]. (**a**) Behaviour of the approach A in the presence of temporary faults in the links. (**b**) Behaviour of the approach B in the presence of temporary faults in the links. (**c**) Behaviour of the approach C in the presence of temporary faults in the links.

As we have anticipated in Section 1, we have designed three different approaches to the PTRF mechanism. The approaches differ from each other in two aspects, (i) what are the devices that carry out the replication of frames and the elimination of surplus replicas, and (ii) what is the technique used to calculate the number of replicas. Figure 3 shows the basic operation of the three approaches in the presence of temporary faults in the links. Note that, even though in this work we assume that our network implements CQF to schedule frames, PTRF is designed to operate with any schedule, not only CQF. For this reason, in our description, we talk about time instants and not windows. All the subfigures depict the same network, which consists of two end-systems and two bridges connected in a line topology. The talker $T_{1}$ transmits a replicated frame in instant $t_{i}$, which is forwarded by the bridge $B_{1}$ in instant $t_{i+1}$ and by bridge $B_{2}$ in instant $t_{i+2}$. Furthermore, during $t_{i+1}$ a temporary fault affects link $l_{2}$ and during $t_{i+2}$ a temporary fault affects link $l_{3}$.

We next explain the main characteristics of the approaches and we describe how each one behaves in the scenario depicted in Figure 3.

#### 4.2.1. Approach A: End-to-End Estimation and Replication

The main characteristic of this approach is that only end-systems replicate frames and eliminate surplus replicas upon reception. Bridges are Commercial Off-The-Shelf (COTS) standard TSN bridges which forward all the frames that they receive, whether they are replicated or not. Furthermore, the number of replicas that talkers must create is calculated to achieve a specific end-to-end reliability, i.e., a given reliability for the largest path from the talker to the listeners considering the unreliability of each one of the devices and links of that path.

As we can see in Figure 3a, the talker $T_{1}$ transmits three replicas of frame $f_{1}$, labelled as $r_{1,1}$, $r_{1,2}$ and $r_{1,3}$. $B_{1}$ correctly receives all replicas and forwards them, but replica $r_{1,1}$ is affected by a temporary fault in link $l_{2}$ and dropped upon reception by $B_{2}$. $B_{2}$ forwards replicas $r_{1,2}$ and $r_{1,3}$, but $r_{1,3}$ is affected by a fault in link $l_{3}$. Thus, listener $L_{1}$ delivers $r_{1,2}$ to the application and drops $r_{1,3}$.

#### 4.2.2. Approach B: End-to-End Estimation, Link-Based Replication

The main characteristic of this approach is that both talkers and bridges replicate frames, whereas both bridges and listeners eliminate surplus replicas. In this sense bridges are not COTS devices, i.e., they also have to implement PTRF. Like in approach A, the number of replicas that the devices must create is calculated to achieve a specific end-to-end reliability. i.e., a given reliability for the largest path from the talker to the listeners considering the unreliability of each one of the devices and links of that path.

In Figure 3b we can see that the talker $T_{1}$ transmits three replicas of frame $f_{1,1}$. $B_{1}$ receives the three replicas correctly, it uses replica $r_{1,1}$ to create a new set of three replicas and ignores replicas $r_{1,2}$ and $r_{1,3}$. Now, $r_{1,1}$ is affected by a temporary fault in link $l_{2}$ and dropped upon reception by $B_{2}$. $B_{2}$ uses $r_{1,2}$ to create a new set of three replicas and ignores replica $r_{1,3}$. Finally, $r_{1,3}$ is affected by a fault in link $l_{3}$ and it is dropped by listener $L_{1}$. Nonetheless, $L_{1}$ correctly receives replicas $r_{1,1}$ and $r_{1,2}$, delivers replica $r_{1,1}$ to the application and ignores $r_{1,2}$

#### 4.2.3. Approach C: Link-Based Estimation and Replication

The main characteristic of this approach is that each port of each talker and bridge may produce a different number of replicas. We must recall that each talker output port in approach A and also each bridge output port in approach B generates the same number of replicas and that this number is calculated to achieve a given reliability for the largest path between a talker and listeners. Conversely in approach C , the number of replicas is calculated independently for each link taking into consideration its unreliability. This allows saving bandwidth, since it makes it possible to reduce the number of replicas in links that exhibit adequate reliability. The main trade-off of approach C is that, like in B, bridges need to implement PTRF.

In Figure 3c we see that, as we anticipated, the number of replicas varies in each link, transmitting three replicas in link 1, two replicas in link 2 and three replicas again in link 3. Specifically, talker $T_{1}$ transmits three replicas of frame $f_{1,1}$, $B_{1}$ receives the three replicas correctly, it uses replica $r_{1,1}$ to create a new set of two replicas and ignores replicas $r_{1,2}$ and $r_{1,3}$. Now, $r_{1,1}$ is affected by a temporary fault in link $l_{2}$ and dropped upon reception by $B_{2}$. Thus, $B_{2}$ only receives $r_{1,2}$ and uses it to create a new set of three replicas. Finally, $r_{1,3}$ is affected by a fault in link $l_{3}$ and it is dropped by listener $L_{1}$. Nonetheless, $L_{1}$ correctly receives replicas $r_{1,1}$ and $r_{1,2}$, delivers replica $r_{1,1}$ to the application and ignores $r_{1,2}$.

### 4.3. Basic Architecture of a PTRF-Enabled Device

When comparing the reliability achievable by different fault-tolerance mechanisms in TSN, it is fundamental to take into account the unreliability of the extra hardware that is needed to implement those mechanisms. In this sense let us refer to talkers, listeners and bridges that include extra hardware components that implement PTRF mechanisms as PTRF-enabled devices.

**Figure 4:**
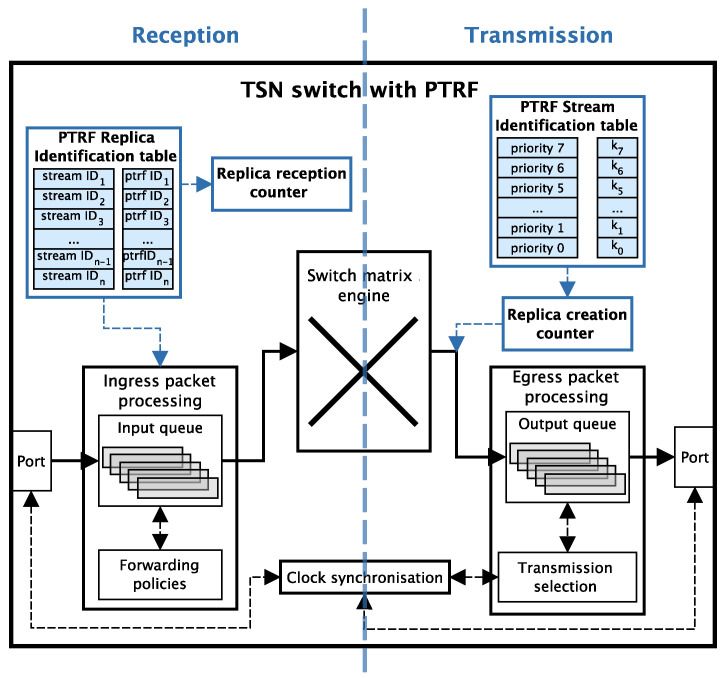
Basic architecture of a TSN bridge with the PTRF mechanism. The additional components PTRF includes are highlighted in blue. Solid lines represent the path frames follow inside the bridge, whereas discontinuous lines represent management or configuration interactions. Reproduced as in [11].

Figure 4 depicts the architecture of a PTRF-enabled bridge, highlighting the extra components it includes when compared with a COTS TSN bridge. This figure also differentiates between the extra components depending on whether they are needed for transmission or reception. In this sense note that PTRF-enabled talkers and listeners also include the extra components of Figure 4 that correspond to the transmission and the reception respectively.

As we can see on the left side of the figure, PTRF relies on two new components to manage replicas upon reception. Specifically, we can see the PTRF Replica Identification Table, in which the bridge or the listener stores information related to the last replica received through each stream. This information is used to eliminate surplus replicas upon reception. The second component is a Replica Reception Counter, which is responsible for monitoring how many correct replicas reach each device for each message edition. In any case, this counter is devised for future extensions of PTRF; e.g., to provide it with the capacity of assessing if the number of generated replicas is enough, or it has to be reconfigured to adapt to an increasingly harsh environment. Therefore, from hereon we will exclude this component from the reliability analysis carried out in this work.

If we now focus on the right side of Figure 4, we see that PTRF includes two additional components to transmit replicated frames. First, the device includes the PTRF Stream Identification Table. This table is needed for knowing which frames must be replicated and how many replicas must be created for each one of them. Furthermore, the device includes the Replica Creation Counter to track the number of replicas it generates for each frame, so that it queues the appropriate number of such replicas in the output queue.

For the sake of succinctness, we do not describe in more detail how these components are used. For a thorough description of the role that each component plays in the operation of PTRF the reader can refer to [11].

## 5. Modelling Rationale

In this section we describe the most relevant aspects of the PRISM models we have developed for TSN and PTRF, namely the reliability metric we use, the fault and the failure models of the PRISM models, the modelling assumptions, the modelling strategy, the model limitations and the model validation. We must indicate that we only develop models for approaches A and B, but not C as it is a specific case of approach B.

We need to keep in mind during the following discussion that, even though PTRF is designed to tolerate faults in the links of the network, we cannot actually quantify the reliability of links as isolated components. Instead, we need to evaluate the reliability that the whole network can reach when implementing PTRF. This is because frames are not only transmitted through links, they also traverse end-systems and bridges. Thus, in order to properly quantify the reliability achievable when implementing PTRF, we need to take into account how PTRF is affected by all the devices of the network.

Another important aspect to keep in mind during the description of the models is that we have developed our models in such a way that, in the future, we can evaluate the reliability achievable when using PTRF together with TSN’s fault-tolerance mechanisms: space redundancy (FRER) [6,7] and error containment [8]. Thus, our models can be tuned to include temporary and permanent faults, as well as time redundancy, space redundancy and error containment. Therefore, even though in this article we focus on temporary faults and time redundancy, we discuss all these aspects of our models.

We next start by defining our reliability metric.

### 5.1. Reliability Metric

In order to build up a model that quantifies the reliability, first we need to particularise this general definition into a reliability metric which the model must calculate. In this sense, the reliability metric must be aligned with the objective of the model. That is, we must decide and state clearly what we are measuring.

It is important to note that we do not model the TSN network of any specific system. Instead, we want to quantify the reliability achievable by *any* TSN network. This implies that the reliability metric has to be defined in such a way that it is general enough to be applicable to the network of any system, regardless of its specific characteristics. Thus, we propose a reliability metric to quantify the reliability of streams.

We must note that in this work we only consider time-triggered traffic. Therefore, in our work a stream is a virtual communication channel that consists of a talker sending a message edition every *T* units of time to one or several listeners through a given number of paths; where *T* is the stream period. In the case of using standard TSN, the talker only sends one copy of each message edition per period (one copy through each path in case of having multiple paths). In the case of PTRF, the talker sends several replicas of each message edition through each path as explained in Section 4.

On top of that, we can specify in the model the number of listeners that must receive each message edition in order for the system to work correctly. In this way, we can take into account systems that only require a subset of the intended end-systems to communicate correctly to provide a correct service, e.g., systems that use node redundancy to tolerate faults and thus can operate with a subset of end-systems.

At this point we can define our reliability metric as the probability that for each stream a subset of the subscribed listeners receives at least one copy of each message edition during a given interval of time called mission time.

In the particular case of the analyses that we show in this article, we do not consider space redundancy. This is so because in this article we want to prevent the reliability benefits of using space redundancy from masking the reliability benefits of the time redundancy provided by PTRF. Thus, our reliability metric is calculated taking into account one path. Furthermore, we only analyse the impact of the reliability for one talker that communicates with a single listener, as this allows us to draw significant conclusions while minimising the factors that can interfere with our analysis. In the future we plan to evaluate networks with several streams, each with several listeners and traffic types.

Finally, it is important to note that this metric alone does not quantify the reliability of a whole distributed system based on TSN. To achieve such a quantification, it would be necessary to model the particularities of a given system, as well as to include all the reliability mechanisms the system is provided with, e.g., the mechanisms to replicate the talker and listener themselves. Thus, our metrics and models have to be understood as a relevant piece that can help in quantifying the reliability of a whole system, but not as the only piece that is necessary for doing so.

### 5.2. Fault Model of the Modelled System

In Section 4 we specified the fault model with which PTRF deals, i.e., temporary non-malicious hardware faults that affect the links. However, we cannot model links as isolated devices, instead, we need to model all the components of the communications subsystem, i.e., links, bridges and the communication controllers of end-systems. Thus, we need to extend the fault model to consider faults affecting the bridges and the communication controllers of end-systems too.

On top of that, we need to take into account that we developed our model taking into consideration TSN’s space redundancy and error contention mechanisms to be able to extend our analyses in the future. Therefore, we need to also include permanent non-malicious hardware faults that affect any device of the network. Therefore, the fault model of the modelled system is permanent and temporary non-malicious operational hardware faults that affect the links, the bridges and the communication controllers of end-systems. This extension of the fault model has resulted in an extension of the failure modes of the system too, which is thoroughly explained in Section 5.3.2.

Particularly, in the analyses presented in this article we have tuned the models to only consider temporary faults that affect links, bridges and the communication controllers of end-systems. As said before, we do not include permanent faults nor space redundancy in the analyses presented in this article as we do not want to mask the benefits that PTRF can yield to the network. Nonetheless, we must note that all the networks that we analyse here include the error-containment mechanisms of TSN. Specifically, our networks include Ethernet’s CRC and TSN’s filtering and policing, which allow the network devices to drop frames that are incorrect in the time and the value domains, as long as these errors are produced at the network level, e.g., a bit flip in the output queue of an end-system or bridge.

Finally, it is important to note that our models do not include faults happening in parts of the system other than the communication subsystem, e.g., software faults and hardware faults affecting the end-system’s main memory. These faults affecting other parts should be considered in the case of modelling a complete system that may include mechanisms to tolerate them; which is not the case of this article.

### 5.3. Modelling Assumptions

Every model relies on a set of assumptions; some of them can be reflected as model parameters, whereas others determine the structure of the model in itself. The model parameters allow us to carry out parametric sensitivity analyses, such as the ones we present in Section 6. As we explain there, first we establish a case of reference, in which we assign to each parameter a value that can be considered as realistic but, at the same time, pessimistic for PTRF. This allows us not to bias this case towards PTRF. Then we carry out a set of analyses; where each one of them consists in quantifying the reliability when varying one of the parameters with respect to the case of reference.

In Section 6 we describe in depth the parameters that we have considered in our models, e.g., the period of the stream, the frame size and the number of replicas, and the range of values we have considered for each one of these parameters in the analyses, their values for the case of reference and their meaning.

In the rest of this section we describe the assumptions that determine the structure of our models; we further comment on the ranges we have considered for some of the parameters. All the assumptions that determine the structure of the model are pessimistic in order to obtain a lower bound to the reliability achievable when using PTRF.

#### 5.3.1. Constituent Components and Failure Rates

To obtain a model that is computationally solvable, it is necessary to abstract away the constituent components of the system to some extent. In our models we differentiate between links, bridges and the communication controllers of the end-systems. For the sake of succinctness, we will refer to the communication controllers as end-systems.

Each component has a different failure rate which varies depending on its complexity and quality. We must note that, since in this work we only consider temporary faults, we only specify the components’ failure rates caused by these faults.

We characterise the rate of temporary faults affecting the links by means of the BER, i.e., the ratio between the number of erroneous bits and the total number of transmitted bits. Specifically, we consider BER values that range from $1\times 10^{-4}$, usually considered in highly critical applications, to $1\times 10^{-12}$, usually considered in non-critical applications [26,27]. In this way we cover BER values considered in relevant applications with a wide range of reliability requirements, e.g., aerospace or automotive [28].

Regarding temporary faults affecting bridges and end-systems, we have considered different failure rates depending on whether they are standard TSN or PTRF-enabled. This is a reasonable assumption since PTRF-enabled devices are slightly more complex, as they count with extra hardware and logic to implement the time redundancy mechanisms, i.e., the ones that support frame replication. This extra hardware is highlighted in blue in Figure 4.

In order to estimate the failure rate of PTRF-enabled devices, we use the implementation of PTRF on a real TSN bridge, which has been implemented using an FPGA as we explain in detail in [11]. Unfortunately, we do not count with the exact information regarding the occupation of this extra hardware in the FPGA. Thus, we make a pessimistic assumption and we estimate that PTRF implies an increase of the 50% in the hardware of the devices and, thus, an increase of a 50% in the failure rate when compared to TSN-enabled devices.

We have considered a failure rate of $1\times 10^{-4}$ per hour for standard TSN bridges and end-systems [29]. On the other hand, we have considered a failure rate of $1.5\times 10^{-4}$ per hour for these devices when they are PTRF-enabled.

#### 5.3.2. Failure Model and Fault-Tolerance Coverages of the Modelled System

The failure model of a system includes the failure modes of each one of its components. Therefore, we need to specify the failure modes of all the devices included in the system, i.e., in this case the devices of the network. The failure modes that constitute the failure model, as well as the proportions with which they manifest, have a strong influence on the system reliability. Thus, we take both these aspects into account while defining our models.

**Figure 5:**
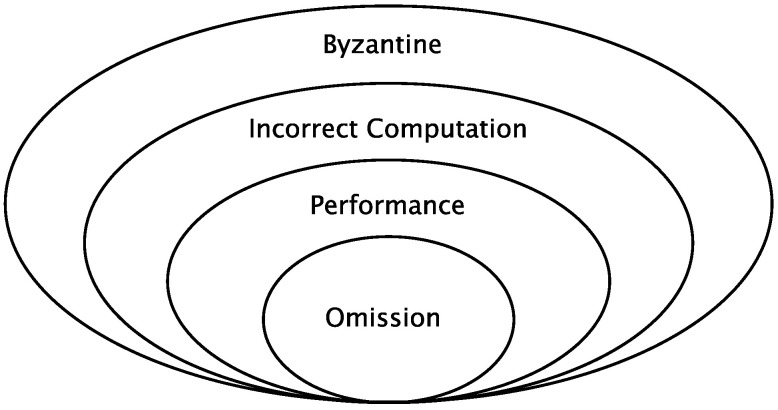
Inclusion hierarchy diagram that depicts the presented failure modes. Figure based on Figure 2.9 in [32].

To better understand the influence of the failure modes, we are going to use a simplified version of the failure mode hierarchy used in [30], which is, at the same time, an extended version of the hierarchy presented in [31]. The simplified version we use in this paper is shown in Figure 5. This hierarchy classifies the failure modes that a system can exhibit in terms of their degree of restriction, being the inner failure modes more restricted, in terms of the faulty behaviours compatible with the mode, than the outer modes. Additionally, inner failure modes are more benevolent and easier to deal with, while outer failure modes are harsher and harder to deal with. We next describe those failure modes relevant to understanding this work:

- Byzantine: lack of restrictions on the way the system can behave. It includes two-faced behaviours, i.e., a faulty device sending different information to different devices, and impersonations, i.e., a faulty device pretending to be a different device.
- Incorrect computation: the system delivers an incorrect result, either in the value or the time domain, but without showing two-faced behaviours nor impersonating other systems.
- Performance: the system delivers a correct result in the value domain, but fails to do it in the time domain.
- Omission: the system delays the delivery of a result forever.

As we have already explained in Section 4, temporary faults affecting a link are assumed to provoke an omission. This means that we will assume that the only failure mode that links exhibit is omission. The consequence of a frame omission on the reliability metric depends on whether the network is provided with space or time redundancy. A frame omission in a given path prevents the listener from receiving the corresponding message edition through that path, unless the frame is replicated in the time domain in that path.

In the particular case of the analyses presented here, we are considering that the network does not have space redundancy, i.e., the stream traverses a single path, both if TSN or PTRF is considered. In the case of TSN, since it does not count with any time redundancy mechanisms, only one copy of each message edition is transmitted through the path. As a consequence, if a temporary fault in a link corrupts a frame, the conveyed message edition does not reach its destination, provoking the failure of the stream. Conversely, PTRF transmits through the path several replicas of the frames that convey each message edition. Thus, the omission of a replicated frame does not provoke the failure of the stream, as long as the listener receives at least one replica of that replicated frame.

As regards bridges and end-systems, they are assumed to exhibit Byzantine failures, when faulty. The impact of such failures on the exchange of frames through the stream depends on the following aspects: (i) whether or not space redundancy is used; (ii) whether the faulty device is TSN or PTRF-enabled; (iii) whether the faulty hardware is involved in the transmission or the reception capabilities of the device, i.e., whether it affects an output or an input port; (iv) how the faulty hardware can affect the transmission/reception operations of the device.

Table 1 specifies the expression used to model the unreliability of bridges and end-systems; the proportions of their different failure modes; and the impact of each failure mode on the reliability metric that we have defined in the previous subsection when using TSN and PTRF with a single path. For instance, we can see that the unreliability of a TSN standard talker is expressed using the equation $1-e^{-(\mathrm{Tx}\times 1\times 10^{-4})}$, where Tx represents the time required for a port to transmit a frame. We can also see that, whenever a fault affects the talker, there is a 50% probability that this fault affects its output port and a 50% probability that it affects its input port. If the fault affects the output port, we see that it can affect the whole port or just the queues and, regardless of the affected component, the fault manifests as Byzantine and, with a 100% probability it results in the failure of the stream.

In the specific case of TSN, when a temporary fault affects the input or the output port of a TSN bridge, the bridge transmits, through one or several output ports, a Byzantine version of the frame it is forwarding. Fortunately, each one of these Byzantine versions is dropped by means of the error-containment mechanisms of the next bridge or listener that receives it; thereby transforming each one of these Byzantine frames into an omitted one. In this sense, the impact of a faulty bridge resembles the one of a faulty link, i.e., it prevents the listener from receiving the affected frames through the corresponding path.

In the particular case of using a single path, this results in the failure of the stream. Conversely, the fault can be tolerated when using multiple paths, as long as the Byzantine failure does not compromise the schedule of other streams transmitted or forwarded by the same device, e.g., by starving the bandwidth of the links connected to the output ports through which the talker or bridge sends Byzantine frames. For the sake of simplicity, we have decided to model this scenario by means of a parameterised probability of affecting the schedule when a Byzantine fault affects a bridge.

A temporary fault affecting a TSN end-system only results in a frame being incorrectly transmitted or received if the fault affects the components involved in the operation of the end-system. Specifically, if the end-system acts as a talker, then the fault can only lead it to transmit a Byzantine version of the frame through a given output port if the fault affects that port. Likewise, in the case of the listener, a fault can only lead it to receive a Byzantine version of the frame through a given input port, if the fault affects that port.

The impact of a Byzantine frame being transmitted by a TSN talker is the same as if it was transmitted by a bridge, i.e., the frame is dropped by the next bridge, causing the stream failure when using a single path or in case the corrupted frame affects the schedule of other streams when using multiple paths. In contrast, we assume that a fault affecting any input port of a TSN listener always results in the failure of the stream. This is because, since the fault happens within the listener itself, it may affect the error-containment mechanisms that supervise the correct operation of the port.

In the analyses we present here, which assume a single path, we consider that each end-system has one output port and one input port. Since the complexity of both ports is similar, we consider that when a fault happens in an end-system, it affects equiprobably its output and input ports. This is shown in the column Prop. of the left side of Table 1, where the proportion with which each port is affected is 50%. In any case, these proportions are parameters of our model that can be tuned to study the impact that modifying this proportion has on reliability.

The analysis of the negative impact of temporary faults affecting PTRF-enabled bridges and end-systems is more complex. Unlike TSN, PTRF does provide time redundancy. Hence, as said before, faults can be tolerated in the corresponding path, as long as the listener receives at least one copy of each message edition through that path. To simplify the explanation from hereon, let us consider a single path. In order to model how faults can be tolerated when using PTRF, we need to differentiate which failure modes PTRF-enabled devices may exhibit on top of the ones exhibited by TSN devices. This is so because certain failure modes may exhaust the time redundancy provided by PTRF and, thus, exceed its fault-tolerance capacity. Table 2 describes the failure modes of PTRF.

For this purpose, let us revisit Figure 4, which shows the extra components of a PTRF-enabled bridge coloured in blue. As already explained in Section 4, the blue components represent the extra hardware required for PTRF to create replicas during transmission and to eliminate surplus replicas upon reception. More specifically, the left side of the figure shows the components that eliminate surplus replicas upon reception. Let us refer to them as PTRF reception components. The right side of the figure presents the components that create and then queue in the output queues the frame replicas. Let us refer to them as PTRF transmission components.

A fault affecting the PTRF transmission components may manifest as the two following failure modes in addition to the Byzantine failure mode of TSN transmission devices: (i) queuing corrupted frame replicas, or (ii) queuing an incorrect number of frame replicas. Table 2 also explains these two failure modes and their impact. The first one provokes the failure of the stream. In contrast, the second one only provokes such a failure when it creates more replicas than expected (n>k), as having more frames in the network would cause the schedule to be violated, or when it creates no replicas (n=0). Otherwise, this second failure mode simply reduces the ability to tolerate further faults affecting the message edition (0<n<k).

The proportion of these PTRF-specific failure modes can be parameterised in the model, but Table 1 shows the proportions selected for our sensitivity analysis. We can see in the table that the proportion of the TSN Byzantine and the buffered failure modes in the output port of PTRF talkers and bridges is 83.33% (labelled as “TSN + buffer” in the table). Let us explain how we obtain this value. As we have previously said, we assume that the failure rate of PTRF devices is 50% higher than for TSN ones due to the extra hardware required by PTRF, i.e., TSN’s logic occupies 2/3 of the hardware while PTRF’s logic occupies 1/3. We then assume that TSN’s Byzantine failure mode has a proportion of 2/3 (66.67%), while PTRF’s failure modes have a proportion of 1/3 (33.33%).

On top of that, we assume that the failure modes of PTRF’s transmission are equiprobable, i.e., the probability of frame replicas being corrupted in the buffer and the probability of creating the wrong number of replicas are the same (50%), and 50% of a proportion of 1/3 is 16.66%.

Since the corruption of frames in the replication buffer always causes the failure of the stream, just like TSN Byzantine failures, we can group these two failure modes into a single failure mode with a proportion of 66.67% + 16.66% = 83.33% (TSN + buffer). On the other hand, since the faults that affect the replica creation counter do not always result in the failure of the stream, we cannot group this failure mode with the ones previously discussed. Since the proportion of faults that affect the replication counter is 16.66% and we assume that the probability of creating more replicas than expected is the same as creating less, we obtain a failure mode proportion of 8.33% for n>k and 8.33% for n<k.

On the other hand, a fault affecting the PTRF reception components may manifest as in TSN, but also in the following two ways: (i) by failing to eliminate all surplus replicas of a message edition upon reception, or (ii) by eliminating all replicas of a message edition upon reception. Table 2 further explains these failure modes and why both of them cause the failure of the stream. As we can see in Table 1, faults in the reception of bridges and listeners always cause the stream to fail. Thus, we group all the failure modes into one which we call fail to eliminate that has a proportion of 100%.

Finally, another important aspect that has an important impact on dependability in general, and on reliability in particular, is the value of the coverage associated with the FT mechanisms. Coverage can be roughly defined as the probability with which a system or fault-tolerance mechanism behaves as intended. We find different types of coverage. First, we find the assumption coverage, which refers to the probability with which faults manifest as stated in the failure model. Second, we find the error containment coverage, which refers to the probability with which an error containment mechanism effectively contains faults.

In our models, the assumption coverage is a parameter that can be modified, but in this article we have established an assumption coverage of 100% for three reasons. First, the assumption coverage of fault-tolerant systems is usually high, as these systems are conscientiously designed to ensure that they exhibit the failure semantics that it is assumed for them. Second, we tend to assume Byzantine failure modes for most devices, which is the least restricted mode and thus corresponds to 100% assumption coverage. Third, we assume that most failure modes cause the failure of the stream, which is a sufficiently pessimistic assumption.

Regarding the coverage of the error containment mechanisms, we must note that the error containment mechanism used to drop erroneous frames is Ethernet’s CRC. As we have discussed in Section 4, the CRC can detect virtually all erroneous frames and, thus, we assume a coverage of 100%. We must highlight that assuming a lower coverage would result in an unfair evaluation of the reliability achievable not only by PTRF but also by TSN.

#### 5.3.3. Topology Assumptions

As we have already explained, we want to evaluate the reliability achievable by TSN and PTRF. Moreover, we want this evaluation to be as general as possible and, thus, we do not model any specific system nor any specific network. Instead, we model the network as a set of streams. Thus, we abstract the network topology away and we only keep in our model those aspects that have a significant impact on the reliability of the stream, e.g., the number of hops that the frames of the stream need to traverse.

We should recall that in Section 3 we have defined a stream as a virtual communication channel which communicates one talker to one or several listeners. Now, we introduce the concept of virtual path, which communicates the talker of a stream to one of the listeners through a number *n* of bridges. Thus, each stream has one virtual path for each listener composed of a talker, a listener and *n* bridges. For simplicity, from now on we will refer to virtual paths simply as paths.

In our models all paths are disjunctive, i.e., the paths do not share any network component. This assumption could be considered optimistic, as it implies that all faults are independent e.g., a fault in an output port of the talker only affects one path of one stream. Nonetheless, we can overcome this limitation by tuning the maximum number of paths that can fail before the stream fails. Thus, we can establish that the failure of one path causes the failure of the whole stream, which is a pessimistic assumption as it means that a fault in one component in one path of a stream affects all the paths of that stream.

In the future we plan to quantify the reliability of specific network topologies, modifying our models to express their particularities. In any case, such quantification is out of the scope of this article.

#### 5.3.4. Traffic Scheduling Assumptions

Since we want to quantify the reliability of TSN and PTRF networks in general, we also want to abstract most details related to the traffic scheduling. Nonetheless, certain aspects related to the traffic are relevant for the design and evaluation of the models. The most important aspect is that we only consider time-triggered periodic messages since in many applications this type of traffic is usually subject to the most stringent reliability requirements. Thus, all the traffic in our model is transmitted periodically and the period is defined in a per-stream manner.

Another important aspect related to the transmission of frames is the passage of time. In Section 3 we propose to use the cyclic queuing and forwarding standard [23] to schedule frames. As we explain in Section 3, CQF schedules frames so that they traverse one and only one hop in each TAS window. Therefore, we have decided to use the TAS window duration as the main time reference for the models. Since the duration of the TAS window in a device depends on the traffic that traverses it, in our model each stream can have a different TAS window duration. Nonetheless, to reduce the complexity of the models we have decided that the TAS window duration must be the same in all the paths of a stream.

Finally, we make two more assumptions related to the traffic. We assume that all the messages fit within one frame, which is a reasonable assumption for most time-triggered traffic with high reliability requirements. Second, the period must always be higher than the end-to-end delay of a frame. All these assumptions prevent us from modelling networks with certain characteristics regarding traffic scheduling. Nonetheless, since we want to measure the reliability of TSN and PTRF networks from a general point of view, these limitations do not reduce the relevance of the results of our comparison. All of these assumptions allow us to model TSN and PTRF approaches A and B using Discrete-Time Markov Chain (DTMC), where the passage of time is associated with the TAS window.

### 5.4. Modelling Strategy

We follow a modular strategy to build our models. Specifically, first we develop a model of the link reliability when transmitting one frame or several replicas of the same frame. The link model allows us to measure the probability of losing a message edition depending on the BER and the number of replicas. Besides the link model, we have developed three different models to evaluate the reliability of TSN, PTRF approach A and PTRF approach B, to which we refer as general models. We use the results of executing the link model to feed the general models. In this way, we simplify the general models and reduce both the state space (preventing it from exploding) and the computation time.

We use PRISM to develop our models. PRISM is a probabilistic model checker that can be used to evaluate the reliability of systems [33]. PRISM allows modelling the system using different stochastic formalisms, among which we find DTMCs. In PRISM the models are developed using a modelling language that allows building a DTMC as a set of modules, which are the main composition units. On top of modules, PRISM allows using variables to define the local state of the modules. The global state of the model is determined by the local state of all the modules of the model. Moreover, PRISM provides commands to specify the behaviour of the modules. PRISM transforms the modules that conform a model into a Markov chain and solves it analytically.

PRISM also provides a property specification language, which allows specifying which properties of the model must be checked. This language can be used to calculate the probability with which the model is in a certain state and, thus, it allows expressing reliability metrics such as the one we have defined in Section 5.1.

For the sake of clarity and succinctness, we do not describe the models in detail. Instead, we next describe the most important characteristics of each model.

#### 5.4.1. Link Model

**Figure 6:**
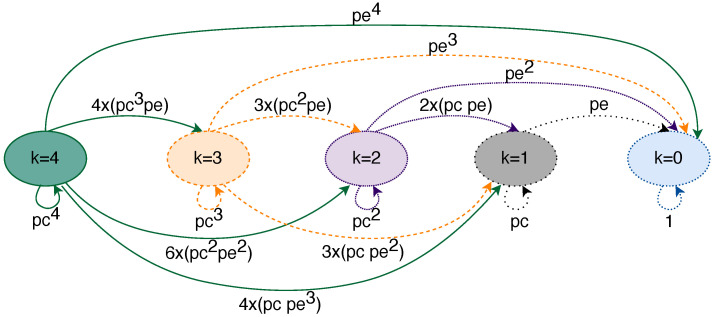
State diagram for the model of a link.

As we have mentioned, we use the link model to measure the probability of receiving a certain number of replicas *n* out of a total of *k* replicas transmitted through a single link with a specific frame size and BER. We must note that, currently, our link model only allows modelling the transmission of *k* replicas, where 1≤k≤4. Figure 6 shows the state diagram of the complete link model. As we can see, the ellipses represent five different states which correspond to the number of replicas that can be transmitted through a link in the model. The transitions between states represent the probability of receiving *n* replicas out of *k*, where n≤k, while they are transmitted through a single link. We must note that the model starts from one of the shown states, depending on the number of replicas we want to transmit. Furthermore, since we evaluate the model for a single link, the model can only reach one state, e.g., if we transmit two replicas, our model starts in the state k=2 and can either stay in the state k=2, move to state k=1 or move to state k=0. We use the same colour and type of line for the initial state and the transitions that the model can take for a single link.

The probability of receiving a subset *n* from a set *k* of replicas depends on the number of replicas that are affected by a fault and can be expressed as follows:

(1) p=kk−n×(1−pe)n×pe(k−n)

where pe is the probability of a frame being affected by a fault. For further details on how we obtain this formula, the reader can refer to [10]. The probability pe of a frame being erroneous is calculated as follows:

(2) $$pe=1-e^{-\frac{BER*framesize}{bandwidth}},$$

where BER is the ratio between the number of erroneous bits and the total number of transmitted bits; framesize is the size of the frame in bits and bandwidth is the bandwidth of the link expressed in bits per second.

As we have mentioned, we use the link model to feed our general models. Thus, in order to be able to model the transmission of any number of replicas (up to four) and the loss of any combination of replicas, we have to execute this model for every value of *k* and *n*, where 0 ≤ *n* ≤ *k*. On top of that, since we carry out a sensitivity analysis, we execute this model for each value of the BER and frame size that we have considered (Table 3). The input parameters of the link model are the following:

- Number of replicas transmitted, *k*. In our model, the minimum value for *k* is 1 and the maximum value for *k* is 4.
- Number of replicas received correctly, *n*. The maximum value of *n* depends on the *k* we define, as we cannot receive more replicas than we transmit. Thus 0 ≤ *n* ≤ *k*.
- Frame size. Necessary to calculate the probability of a frame being affected by a fault, as depicted in Equation (2). We use the frame size values set for our sensitivity analyses: 64, 782 and 1500 bytes.
- BER. Necessary to calculate the probability of a frame being affected by a fault, as depicted in Equation (2). We evaluate the BER values set for our sensitivity analyses: from $1\times 10^{-4}$ to $1\times 10^{-12}$.

These experiments resulted in a total of 378 executions of the link model.

**Table 3:** Parameters tuned in the the sensitivity analysis.

Parameter	Range	Reference	Meaning
Mission time	10 h	10 h	The time that the system is expected to operate in a continuous manner. We use 10 h in all the experiments as it is typically assumed for evaluating the reliability of critical applications such as throttle-by-wire [34].
TAS window	1.3 ms	1.3 ms	The duration of the TAS window in milliseconds. Since we assume that frames are scheduled using CQF, each TAS window must last enough time for a frame to be transmitted, propagated, received and forwarded. Thus, to decide on the TAS window size we have taken into account the transmission and reception time, the propagation time and the forwarding time of a frame through a link and a bridge. These values have been mathematically estimated or measured using a real TSN bridge [35]. This parameter is fixed in all the experiments.
Period	15 TAS windows	15	The number of TAS windows that elapse between the transmission of frames. This parameter is fix in all the experiments.
BER	{$1\times 10^{-4}$, ..., $1\times 10^{-12}$}	$1\times 10^{-10}$	The bit error rate of the links. We consider values from $1\times 10^{-4}$ considered in highly critical applications such as space missions, to $1\times 10^{-12}$ considered for utility communications. We use $1\times 10^{-10}$ as reference as it is the most pessimistic assumption while still considering critical applications such as automotive.
Frame size	{64, 782, 1500}B	782	The size of the frames transmitted through the stream in bytes, being 64 the minimum size, 782 the medium size and 1500 the maximum size for Ethernet frames. We use 782 as reference to compare the impact when using smaller and larger frames.
# of replicas	{1, ..., 4}	2	The number of replicas transmitted by PTRF-enabled components. This value is always 1 in TSN and we consider up to 4 replicas as our results show that reliability converges with this number of replicas. Our case of reference is 1 replica for TSN and 2 replicas for PTRF as this is the lowest number of replicas that PTRF can transmit while still providing time redundancy.
# of bridges	{2, ..., 10}	6	The number of bridges in the path between the talker and the listener. We consider from 2 to 10 bridges. We select 6 bridges for our case of reference as this is the maximum number of bridges for which TSN ensures tight synchronisations.

#### 5.4.2. General Models

**Figure 7:**
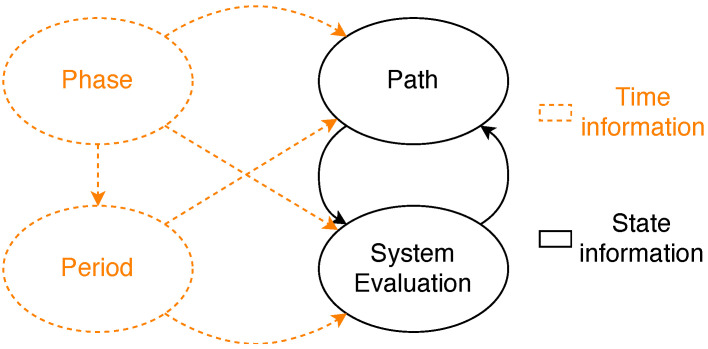
Modules in the general model. The module system evaluation decides whether the system has failed, the module path models the transmission of frames, and the modules phase and period manage the pass of time in the model.

As we have mentioned, we have developed three different general models, one for standard TSN, one for approach A and one for approach B. Nonetheless, all the models share the same structure presented here. Figure 7 shows the different modules that make up the models and how they interact. Specifically, each model has a system evaluation module, a path module, a phase module and a period module. The orange dashed lines represent time information, while the black solid lines represent information related to the state of the model. We next describe each module in more detail.

**Figure 8:**
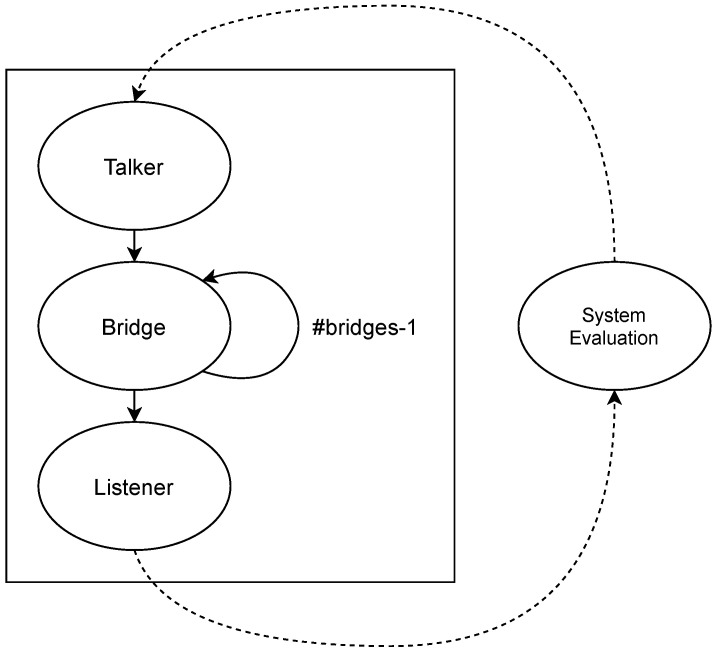
Blocks that constitute the path module.

**Figure 9:**
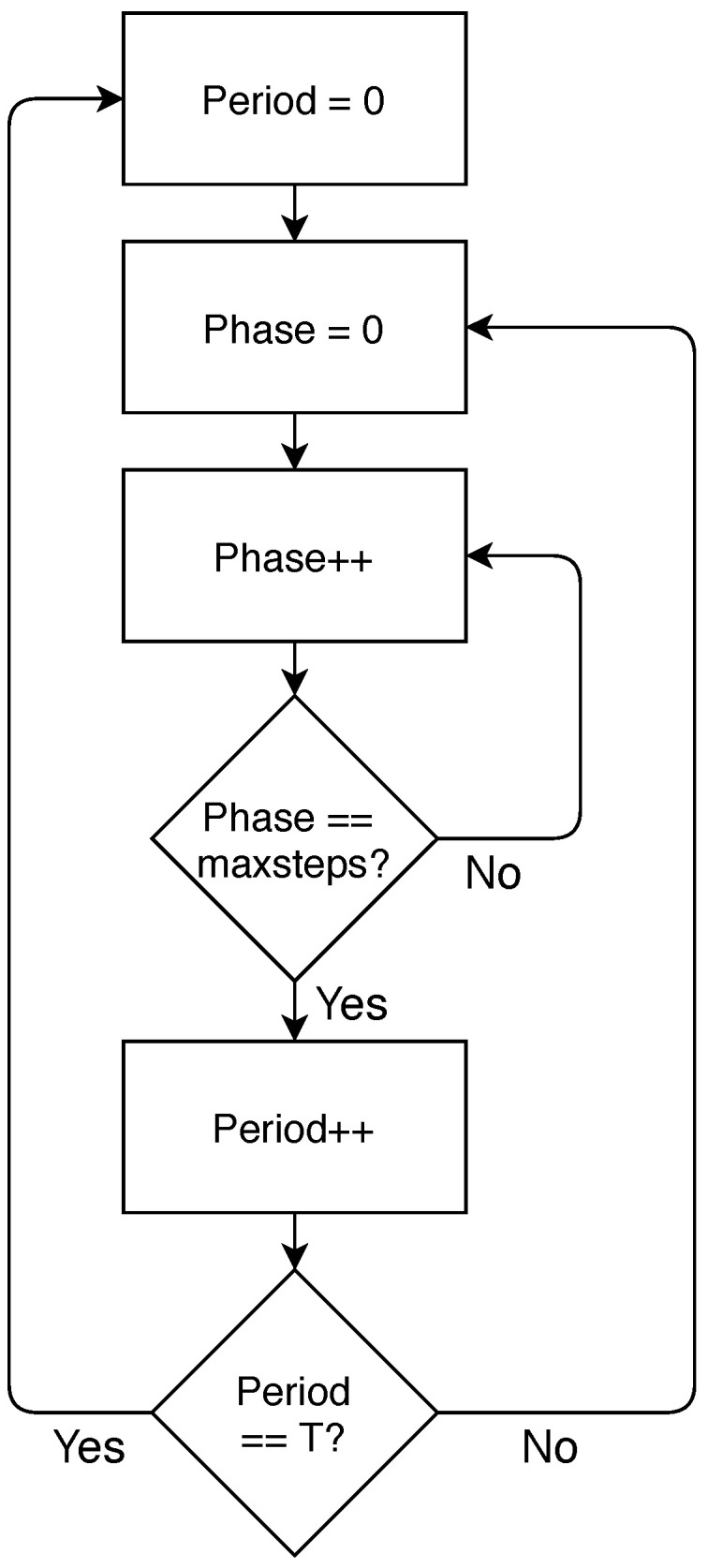
Execution of the phase and period modules to dictate the pass of time in the models.

- Module system evaluation. This module decides whether the system has failed according to a set of predefined rules which are applied to the local variables of the path, phase and period modules. Specifically, the system fails if *too many* streams fail; a stream fails if *too many* paths fail, and a path fails if all the replicas of a message edition are lost in the path. The result of this module is shared with the path modules so that the affected path modules stop evolving (i.e., making calculations) when the system has already failed. Whenever the number of failures in any of these modules reaches the specified threshold, the whole model stops.
- Module path. This module models the transmission of frames through a given path of a stream. Specifically, for each message edition this module models whether the transmission, forwarding and reception of each replica are correct or not for all the hops of the path. If a stream has *L* intended listeners there must be *L* path modules for said stream. This module is further divided into three blocks, namely talker, bridge and listener. Figure 8 shows the different blocks that constitute the path module and their relationships.
  As we can see in Figure 8, the execution of the path module starts with the talker block, which transmits a frame or the specified number of replicas of a frame every period.
  The bridge block evaluates how many frames transmitted by the talker or the preceding bridge successfully reach the destination bridge of the current hop. Specifically, the bridge uses the loss probabilities calculated using the link model previously described to carry out this evaluation. Then it evaluates if the bridge correctly forwards the adequate number of frame replicas (or a single frame in TSN) following the corresponding approach depending on the model: TSN, PTRF approach A, or PTRF approach B. In case of deciding that the bridge fails in the forwarding, the bridge block models the specific way in which this failure manifests, e.g., by forwarding more replicas than expected. The bridge block is executed as many times as bridges are in the path.
  The listener block evaluates how many frames transmitted by the last bridge of the path successfully reach the listener. Then, it decides if the listener correctly eliminates the surplus replicas in the case of PTRF and if it correctly delivers the frame to the upper layer. Once the path is completed, the system evaluation module determines whether the stream is faulty or not and, if not, the path module is executed again when dictated by the period module.
- Module phase. We have just explained, the path module is divided intro three different blocks, namely the talker, the bridge and the listener block. Additionally, each block executes several steps, e.g., the bridge block receives, forwards and transmits frames. We must recall that we use CQF for the scheduling of frames, which means that all the steps of one block are executed within the same TAS window. The phase module dictates when the different steps of a block must be done within the TAS window.
  We must note that not all blocks execute the same number of steps. More concretely, the bridge is the block with the highest number of steps. Nonetheless, we have assumed that all the TAS windows have the same duration for a specific stream and, thus, the number of phases is the same for all the blocks of the stream. When the talker and the listener complete all the steps, they remain idle until all the phases pass, just like a real TSN device would do.
- Module period. This module dictates when each block of the path module is executed, i.e., it dictates the pass of time in a stream. Since we use CQF to schedule frames, each block of the path module is executed during one TAS window. The module period also counts the number of TAS windows in a period of a stream and triggers the transmission of a frame in the talker when required.
  Figure 9 shows the relationship between the phase and period modules. Specifically, the execution of the complete model starts initialising the period and phase modules to 0. The phase module is increased as many times as steps executed the bridge (as previously explained). Once all the steps of a block are executed, the period is increased and the phase module is reset.
  If the period is higher than the number of hops, the execution of the path module is completed before the period module reaches the specified period *T*. In that case, the path module remains idle until the period module reaches the value specified for the streaming period, then the period is reset and the talker creates a new frame.

### 5.5. Model Testing

We check that the models have been correctly implemented using two different strategies. On the one hand, we use the simulation tool available in PRISM, which allows tracing the execution of the model. First, we use the simulation tool to execute the model step by step. This allows us to detect problems and correct them so that the models operate as intended. Second, we use the simulation tool to execute several steps of the model at once to detect interlocks, i.e., we execute the models in sets of 1000 steps at a time, which allows exploring the model faster.

On the other hand, we have executed the models using different configurations. Specifically, we have assessed the proper execution of the model using (i) a single stream with a single path; (ii) a single stream with up to five paths; (iii) up to three different streams, each with up to three paths and (iv) replicated streams with one path each, allowing us to model space redundancy.

The results of the aforementioned tests allowed us to refine the implementation of the models to eliminate mistakes, improve their efficiency in terms of execution time and validate their correctness.

## 6. Parameters of the Models

In order to carry out the comparison between TSN, approach A and approach B, we must first define a case of reference that serves as the baseline to compare the results of the models, as well as to study the impact of the different parameters on reliability. To that end, we assign a reference value to each parameter and we define the range of values that each parameter takes. We must note that the case of reference must have reasonably realistic values for the parameters, while still being pessimistic for PTRF. In this way, we do not bias the results in favour of PTRF.

Table 3 shows the parameters that we have tuned during the analysis, the range of values that we have evaluated as part of the sensitivity analysis, the value we have assigned for the case of reference and a brief description of the parameter. The case of reference is highlighted in bold.

As we can see, our models have seven different parameters that can be tuned during the analysis. We have fixed three parameters, namely the mission time, the duration of the TAS window and the period of the stream. On the other hand, we vary and thus evaluate the impact of four different parameters, namely the BER, the frame size, the number of replicas and the number of bridges.

## 7. Results

We have executed an experiment for each combination of parameters, which has resulted in 2146 experiments. In each one of these experiments, we have modelled the transmission of 1,800,000 frames. We next analyse the most relevant results obtained, starting by the case of reference and moving to the analysis of the impact that each parameter has on the reliability.

### 7.1. Case of Reference

As we have explained at the beginning of this section, we have established a case of reference to compare the different solutions and the impact that the different parameters have on the reliability. We must note that, as we can see in Table 3, the number of replicas transmitted by PTRF in the case of reference is two, as this is the lowest number of replicas that PTRF can use while still providing time redundancy. Later on, we show how transmitting a single frame when using PTRF affects the reliability.

Table 4 shows the results obtained in the case of reference for TSN, approach A and approach B. As we can see, the results obtained with all the solutions are in the same order of magnitude. However, TSN can achieve a reliability of 85.84%, while approaches A and B both reach a reliability of 99.99% when transmitting just two replicas.

If we now focus on PTRF, we can see that approach A is more reliable than approach B. This is because in approach A only the talker and the listener are PTRF-enabled devices and bridges are standard TSN devices with lower failure rate; unlike approach B where all the devices are PTRF-enabled. Since the BER in the case of reference is low, the contribution of the failure rate of the devices in the unreliability is higher than in networks with larger BERs and, thus, the unreliability of bridges is more noticeable.

From this first set of results, we can extract several conclusions. First, we can conclude that the use of time redundancy has a positive impact on the reliability achievable in networks that suffer temporary faults. Second, we see that the benefits of time redundancy are even noticeable in networks with a relatively low BER, only $1\times 10^{-10}$, and with a relatively small network, only six bridges. Finally, we can see that approach A is a more adequate solution than approach B in networks with low BER, as the contribution of the failure rate of the components increases the unreliability in the latter.

In the following subsection we analyse how the BER affects reliability and we pay special attention to higher BER values.

### 7.2. Bit Error Rate

**Figure 10:**
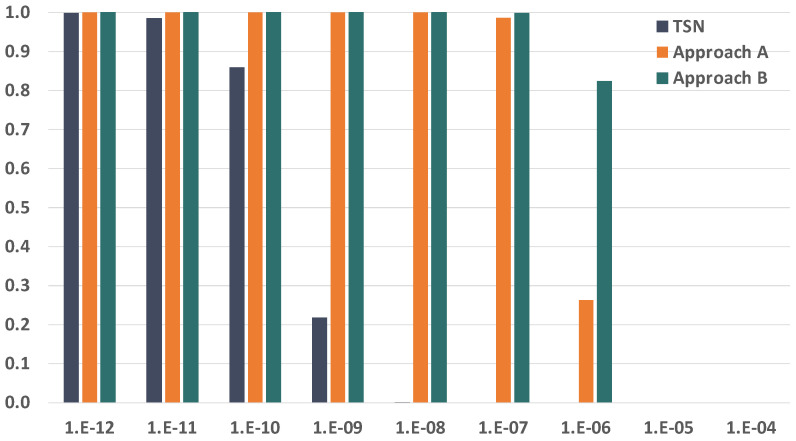
Reliability achievable when varying the BER from $1\times 10^{-12}$ to $1\times 10^{-4}$. TSN is represented in gray, approach A in orange and approach B in green. The *x* axis represents the BER values, while the *y* axis represents the reliability.

**Figure 11:**
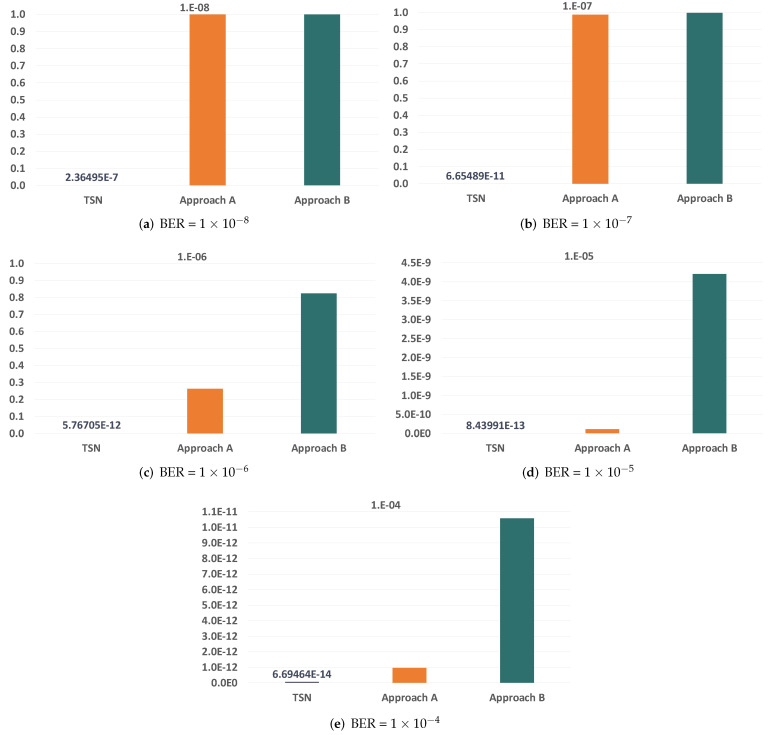
Zoom in the reliability achievable by TSN, approach A and approach B for BERs from $1\times 10^{-8}$ to $1\times 10^{-4}$. TSN is represented in grey, approach A in orange and approach B in green. The *x* axis represents the BER values, while the *y* axis represents the reliability.

Since we are evaluating the impact of temporary faults in the network, the first parameter we want to study is the BER of the links. The BER of the links severely affects the reliability of the communication subsystem, so we have selected a wide range of BER values to carry out our analysis, which go from $1\times 10^{-12}$ to $1\times 10^{-4}$. Figure 10 and Figure 11 show the evolution of the reliability achievable by TSN, approach A and approach B when modifying the BER.

As expected, we see that the reliability is severely affected by the increase in the BER. We can also see that the impact is significantly higher in TSN than in approaches A and B when the BER is the same. This is because TSN does not count with any time redundancy mechanism to tolerate the faults in the links, while approaches A and B do. Furthermore, these results also show that approaches A and B can reach reliability values over 99.9% for BER values up to $1\times 10^{-8}$ and over 99.8% for a BER of $1\times 10^{-7}$ when transmitting just two replicas. As the BER increases, we see that the reliability drops dramatically even when using PTRF. This is because two replicas are no longer enough to tolerate the great number of faults that affect the links in those cases.

If we pay close attention to the results of approach A and B, we see that the increase in the BER has a higher impact in approach A than in approach B. This is because in approach A there is a single set of replicas that traverse the network, so whenever a replica is affected by a fault in a link it is lost in the whole path. On the contrary, in approach B bridges create a new set of replicas in each hop, so even if a replica is affected by a fault in a link the following bridge transmits two replicas again. Therefore, we can conclude that in networks with high BER values, approach B is a more adequate solution as it can provide higher reliability values than approach A using the same number of replicas and, thus, the same bandwidth.

These results confirm the benefits of using time redundancy to tolerate temporary faults and show that in many cases a low level of redundancy can provide benefits. Nonetheless, we can see that two replicas are not enough to reach the reliability levels required in highly critical applications, e.g., 99.999% for throttle-by-wire applications [34].

Finally, the huge difference in the reliability achievable when moving from a BER of $1\times 10^{-7}$ to $1\times 10^{-6}$ can let us envision that the BER value can impact the results obtained when studying how the frame size, the number of replicas and the number of bridges affect reliability. Thus, if we only study the behaviour of PTRF in scenarios with BER of $1\times 10^{-10}$, we could be masking important interactions between parameters. Therefore, we have expanded the case of reference to include a BER value of $1\times 10^{-6}$, which is high enough to see how PTRF behaves in harsh environments.

### 7.3. Frame Size

**Figure 12:**
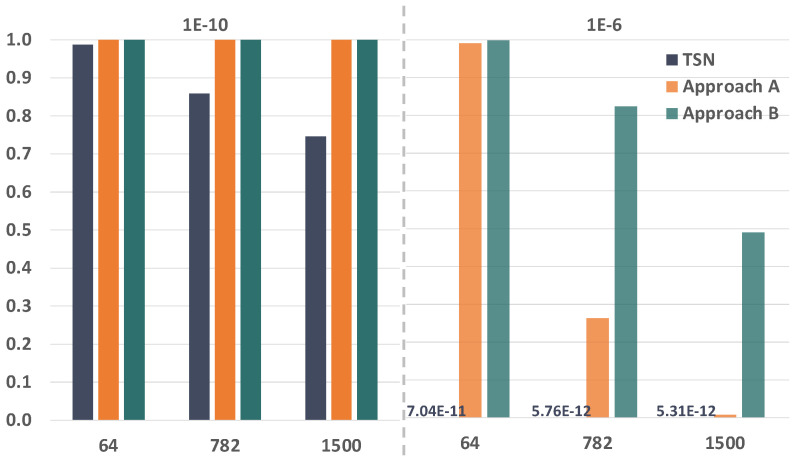
Reliability when varying the frame size. The left side of the figure shows the reliability for a BER of $1\times 10^{-10}$, while the right shows the reliability for a BER of $1\times 10^{-6}$. In both cases, TSN is represented in grey, approach A in orange and approach B in green. The *x* axis represents the frame sizes in bytes, while the *y* axis represents the reliability.

A temporary fault in the communication subsystem only results in an error if a frame is being transmitted while the fault happens. Therefore, we can assume that the size of the frame impacts the reliability, as larger frames occupy the communication subsystem for a longer time than smaller frames. The following experiments allow us to quantify this impact. Figure 12 shows the reliability achievable by TSN, approach A and approach B when transmitting frames of 64, 782 and 1500 bytes in two cases, namely, our case of reference with BER equal to $1\times 10^{-10}$ and another case with a BER of $1\times 10^{-6}$.

If we look at the results obtained when the BER is $1\times 10^{-10}$, we can see that the frame size has an important impact on the reliability achievable by TSN, which drops more than a 20% with larger frames. In contrast, we can see that in this case the impact for approaches A and B is negligible. This shows that, when the BER is low, using the minimum level of temporal redundancy (number of proactive replicas, *k*, equal to 2) is enough to compensate the negative impact of using large frames. Moreover, the results of approach A further indicate that bridges do not need to regenerate replicas at their output ports (as done in B) to compensate this negative impact; at least when the path does not include more than six bridges.

This benefit of temporal redundancy is corroborated, to some extent, when we consider a BER of $1\times 10^{-6}$. However, in this case, the reliability of A and B drops around 98% and 50% respectively when the frame size is 1500 bytes instead of 64. This indicates that when the BER is high, it is necessary to increase the number of time replicas to keep a high level of reliability; especially when using approach A instead of B. Later on, we demonstrate this assertion (for the reference frame size of 782 B) when analysing the impact of the number of replicas.

As a general recommendation, we can say that in critical applications that operate in harsh electromagnetic environments, it may be convenient to reduce the size of frames whenever possible. Nonetheless, this is not always possible. In those cases, approach B is a more adequate solution than approach A, but even with approach B the reliability decreases dramatically. Thus, in such environments larger frames should have a higher level of redundancy than shorter ones to provide adequate levels of reliability.

### 7.4. Number of Replicas

It is expected that the number of replicas transmitted for each frame impacts the reliability achievable by the communications subsystem. Nonetheless, we need to note that the level of time redundancy can negatively impact other aspects of the system, such as the end-to-end delay, jitter and bandwidth consumption, as we show in [11]. Therefore, choosing a level of temporal redundancy that provides the network with the intended reliability, while ensuring an adequate quality of service is of utmost importance.

**Figure 13:**
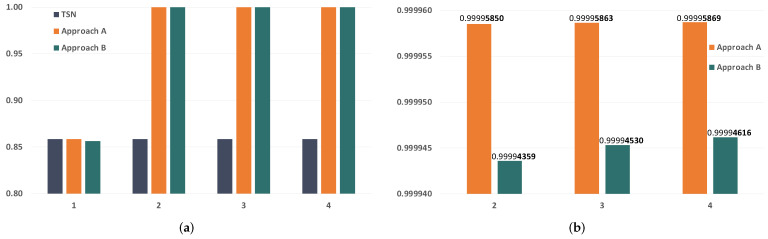
Reliability achievable when varying the number of replicas in a network with a BER of $1\times 10^{-10}$. TSN is represented in grey, approach A in orange and approach B in green. The *x* axis represents the number of replicas, while the *y* axis represents the reliability. (**a**) Reliability achievable by TSN, approach A and approach B when varying the number of replicas. In TSN the number of replicas is always 1. (**b**) Zoom in the reliability achievable by approach A and approach B when varying the number of replicas.

Figure 13 shows how the number of replicas transmitted impacts the reliability achievable by TSN, approach A and approach B when the BER of the network is $1\times 10^{-10}$. Note that the number of replicas in TSN is always one, as it does not count with any time redundancy mechanism.

If we focus on Figure 13a what we first see is that the reliability is improved by almost 15% when using time redundancy in this scenario. However, if we focus on the first three columns, when replication is not used, we can see that the reliability actually drops when using approaches A and B to transmit non-replicated frames. This is because PTRF-enabled devices have a higher temporary failure rate ($1.5\times 10^{-4}$) than standard TSN devices ($1\times 10^{-4}$). Thus, when no redundancy is used, the probability of losing frames due to temporary faults affecting the bridges or end-devices is higher in PTRF than in TSN. Furthermore, this is more noticeable in approach B than in approach A, as the number of PTRF-enabled devices is higher in the former.

In fact, if we focus on Figure 13b we can see that the increased failure rate of PTRF-enabled devices limits the reliability benefits provided by B when compared to A. This is because PTRF is designed to tolerate link faults only and, thus, it cannot tolerate most faults that occur in end-systems or bridges. Therefore, in networks where the BER is low, approach A is a more adequate solution than approach B to tolerate temporary faults. In any case, we see that the reliability is always above 99.99% for both approaches.

**Figure 14:**
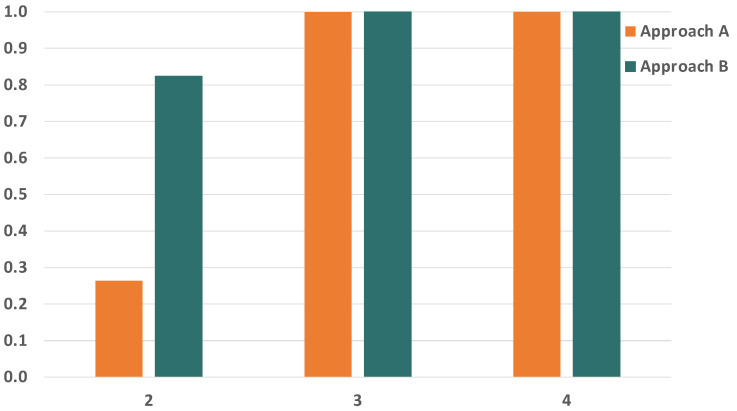
Reliability achievable when varying the number of replicas in a network with a BER of $1\times 10^{-6}$. Approach A is represented in orange and approach B in green. The *x* axis represents the number of replicas, while the *y* axis represents the reliability.

On the other hand, Figure 14 depicts the reliability achievable by approach A and approach B when transmitting a different number of replicas in a network with a BER of $1\times 10^{-6}$. Please note that we do not include k=1 in this plot as the reliability achievable by the three solutions in the absence of redundancy is around 5.76 × 10^−12^. As we can see, the reliability increases significantly with the number of replicas transmitted. As indicated in the section devoted to analyse the frame size, we also see that transmitting only two replicas is no longer enough to achieve high reliability, especially in approach A, where the reliability is only 26.31%, compared to the 82.36% of approach B.

Furthermore, we can see that the comparison of the reliability benefits provided by A versus B is the opposite of what we have just described when considering a low BER of $1\times 10^{-10}$. Specifically, we can see that when the BER is high approach B provides higher reliability than approach A, even with a low number of replicas. This is because when the BER is high, its contribution to the unreliability is higher than the contribution of the failure rate of devices, i.e., it is more likely that a frame is affected by a fault in the link than in a device. Thus, even though approach B has more PTRF-enabled devices with higher failure rate than approach A, approach B is a more adequate solution than approach A in networks where the BER is high.

Finally, please recall that these results for a BER of $1\times 10^{-6}$ demonstrate (for a frame size of ≤782 B) what we anticipated in the section that analyses the impact of the frame size, i.e., that to keep a high level of reliability when the BER is high, it is necessary to increase the number of time replicas; especially when using approach A instead of B.

### 7.5. Number of Bridges

**Figure 15:**
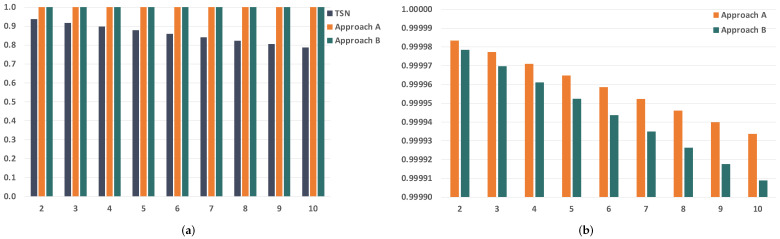
Reliability achievable when varying the number of bridges in a network with a BER of $1\times 10^{-10}$. TSN is represented in grey, approach A in orange and approach B in green. The *x* axis represents the number of bridges, while the *y* axis represents the reliability. (**a**) Reliability achievable by TSN, approach A and approach B when varying the number of bridges. (**b**) Zoom in the reliability achievable by approach A and approach B when varying the number of bridges.

The size of the network is expected to impact the reliability of streams, as in a larger path there are more chances for a frame to be affected by a fault. Figure 15 shows the reliability achievable by TSN, approach A and approach B in networks of different sizes and a BER of $1\times 10^{-10}$.

As expected, we can see that the reliability decreases as the network size increases. If we look at Figure 15a we see that the impact is higher in TSN than in approaches A and B, as TSN does not count with time redundancy. In any case, we can see that the reliability drops around 15% for TSN when moving from 2 to 10 bridges. If we compare this results to the ones obtained previously, we can conclude that in TSN networks with low BERs the impact of the network size is lower than the impact of other parameters.

If we now focus on Figure 15b we see that the reliability using approach B is lower than using approach A. We also see that the difference in terms of this negative impact between both approaches increases with the number of bridges. To understand this effect please recall that the bridges of approach B are PTRF-enabled devices and, thus, they have a higher failure rate than the bridges of approach A. Additionally, recall that this does result in approach A being more reliable than approach B when the BER is low. In this sense, what we observe in Figure 15b indicates that, when the BER is low, the negative impact of using PTRF-enabled bridges instead of TSN ones increases with the size of the path, as a larger path includes more bridges. In any case, we must note that the reliability achievable by PTRF is above the 99.99%, and higher than in TSN, when the BER is $1\times 10^{-10}$.

**Figure 16:**
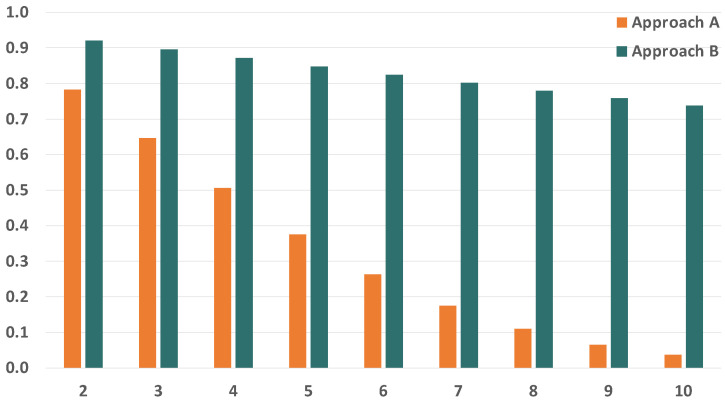
Reliability achievable when varying the number of bridges in a network with a BER of $1\times 10^{-6}$. Approach A is represented in orange and approach B in green. The *x* axis represents the number of bridges, while the *y* axis represents the reliability.

Finally, Figure 16 shows the reliability achievable by approach A and approach B in networks of different sizes with a BER of $1\times 10^{-6}$. We must note that we do not plot the reliability of TSN in this scenario as it is too low to be depicted together with approach A and B (under $5.8\times 10^{-12}$). Again, we can see that the reliability is affected by the size of the network. Furthermore, we see that the higher the BER is, the higher the impact will be.

However, conversely to what we observe in Figure 15b, we see that approach B is a more adequate solution than approach A in networks with higher BER values. In fact, the difference of reliability of approach B when compared to approach A increases with the size of the path. This does not only reinforce the result we showed before about the higher reliability of B when compared to A when the BER is high, but it also shows that, when the BER is high, the positive impact of the additional degree of time redundancy provided by PTRF-enabled bridges further counterbalances the negative impact of their higher failure rate as the path size increases. Specifically, we see that the reliability achievable by approach B drops 18% while the reliability achievable by approach A drops as much as 74%.

## 8. Conclusions

The TSN TG has been working to provide standard Ethernet with real-time guarantees and reliability in layer 2 of the network architecture. These new capabilities are especially relevant for applications such as automotive, industrial automation and aerospace, as these applications are considered to be critical since their failure could have catastrophic consequences in terms of lives and cost. Therefore, these applications must provide a correct service in a continuous manner during their mission time. To that, the systems that support said applications must be reliable in all the levels of the architecture, including the communication subsystem.

The TSN TG proposes two fault tolerance mechanisms to increase the reliability of Ethernet networks. On the one hand, the TG has developed a space redundancy mechanism which is standardised in [6,7] which allows transmitting several copies of each frame in parallel through different physical paths. On the other hand, TSN has standardised an error containment mechanism [8] to reduce the negative impact of incorrect frames in the network.

Nonetheless, the TSN TG does not propose any time redundancy mechanisms to tolerate temporary faults in the links. This is especially important as temporary faults in the links are the most common type of fault that can affect the communication network. Even though temporary faults could be tolerated using space redundancy, this is not a cost-effective solution, as space redundancy requires the addition of hardware which increases the cost, size, weight and energy consumption of the system. Instead, temporary faults can be tolerated using time redundancy.

In previous works we proposed the PTRF mechanism to provide time redundancy to TSN-based networks. PTRF consists in transmitting several copies of each frame in a preventive manner to increase the probability of at least one copy reaching its destination even in the presence of temporary faults. In this work, we carry out a parametric sensitivity analysis of the reliability achievable by TSN and PTRF, in order to assess the benefits that PTRF can yield to TSN networks. To that end, we model TSN and PTRF using PRISM, a probabilistic model checker tool that allows evaluating the reliability of systems using discrete-time Markov Chains, among other techniques.

In this work we have seen that the reliability achievable by any system or subsystem is affected by a myriad of aspects. Therefore, there is not a single solution that can fulfil the reliability needs of any system. Instead, an adequate specific solution must be used for each system. This solution must be configured appropriately in order to provide the required level of reliability, especially in critical systems that operate in harsh environments. The analyses herein presented can help designers in making better decisions when building the communication subsystem of TSN networks.

On the one hand, we have corroborated the idea that the BER is determinant in the reliability achievable by TSN, PTRF approach A and PTRF approach B. Furthermore, we have seen that approach A is a more adequate solution than approach B to provide high reliability in networks with low BERs. Not only does approach A provide a higher reliability level, but it is also a cheaper solution as it uses COTS TSN bridges. On the contrary, approach B can reach significantly higher levels of reliability than approach A in networks with high BERs. Thus, in these cases using approach B is more adequate than using approach A, even if the cost of the network is higher.

Furthermore, we have seen that the frame size has an important impact on reliability, which increases significantly as the BER increases. Therefore, the size of the frames should be reduced whenever possible, e.g., preprocessing the information in the source. If this is not possible, streams that convey larger frames should have a higher level of redundancy than those that convey smaller frames when high reliability is required.

We have also studied how network size impacts reliability. Specifically, we have seen that the reliability decreases as the network size increases. We have also seen that this negative impact increases with the BER.

Finally, we can state that these analyses prove that we can increase the reliability of networks based on TSN by using proactive time redundancy to tolerate temporary faults in the links.

## Figures and Tables

**Table 1:** Unreliability of each device, failure mode proportions and their impact on the stream.

Device	Unreliability	Faulty Port	Prop.	Faulty Component	Failure Mode	Prop.	Stream Failure
TSN Talker	1 − $e^{-(\mathrm{Tx}\times 1\times 10^{-4})}$	Output	50%	Port/queue	Byzantine	100%	**Y**
		Input	50%	-	-	-	**N**
TSN Bridge	1 − $e^{-(\left(\mathrm{Rx}+\mathrm{Fw}+\mathrm{Tx}\right)\times 1\times 10^{-4})}$	-	100%	-	Byzantine	100%	**Y**
TSN Listener	1 − $e^{-(\mathrm{Rx}\times 1\times 10^{-4})}$	Output	50%	-	-	-	**N**
		Input	50%	Port/queue	-	100%	**Y**
PTRF Talker	1 − $e^{-\left(\mathrm{Tx}\times 1.5\times 10^{-4}\right)}$	Output	50%	Port/queue	TSN + buffer	83.33%	**Y**
				PTRF transmission	counter > *k*	8.33%	**Y**
					counter < *k*	8.33%	($\frac{k-1}{k}$)**N**/($\frac{1}{k}$)**Y**
		Input	50%	-	-	-	**N**
PTRF Bridge	1 − $e^{-(\left(\mathrm{Rx}+\mathrm{Fw}+\mathrm{Tx}\right)\times 1.5\times 10^{-4})}$	Output	50%	Port/queue	TSN + buffer	83.33%	**Y**
				PTRF transmission	counter > *k*	8.33%	**Y**
					counter < *k*	8.33%	($\frac{k-1}{k}$)**N**/($\frac{1}{k}$)**Y**
		Input	50%	PTRF reception	Fail to eliminate	100%	**Y**
PTRF Listener	1 − $e^{-(\mathrm{Rx}\times 1.5\times 10^{-4})}$	Output	50%	-	-	-	**N**
		Input	50%	PTRF reception	Fail to eliminate	100%	**Y**

Tx: time required for the port to transmit the frame. Rx: time required for the port to receive the frame. Fw: time required for the bridge to forward a frame from the input port to the output port. -: the value is irrelevant as it does not affect the result.

**Table 2:** Failure modes of PTRF-enabled devices and how they affect the overall system.

Component Failure	Description	Stream Failure
Frames corrupted in the replication buffer	Temporary faults can result in the corruption of frames before they are replicated, e.g., due to a bit flip. If the frame that is going to be replicated is corrupted, all the replicas will be corrupted too and the receiver will drop them when checking the CRC. If this happens the intended listeners do not receive the frame and, thus, they cannot carry out their operation properly.	Yes
Frame replication counter corrupted, creating *n* ≠ *k* replicas.	*n* > *k*. If a device creates more replicas than expected their transmission may affect the transmission of correct scheduled frames.	Yes
	0 < *n* < *k*. If the device creates fewer replicas than expected, then it provokes redundancy attrition, i.e., it reduces the number of forwarded frame replicas and thus the capacity of tolerating faults of the corresponding message edition in the hop, but does not provoke the failure of the stream.	No
	*n* = 0. If the device does not send any replicas of the frame, the intended listeners do not receive the frame and, thus, they cannot carry out their operation properly.	Yes
Failing to eliminate surplus replicas upon reception	A PTRF device can fail to eliminate all surplus replicas upon reception. If this happens in a bridge, the surplus replica will be also forwarded and transmitted, violating the schedule. Let us illustrate this with an example. A PTRF bridge receives the first replica of a frame *i* $r_{1,i}$, it correctly forwards replica $r_{1,i}$ and uses it to create a new set of replicas. Later on, the same bridge receives replica $r_{3,i}$, and it fails to eliminate it. Thus, the bridge forwards replica $r_{3,i}$ and uses it to create a new set of replicas again, resulting in the transmission of twice the number of expected replicas and interfering in the transmission of correct frames. On the other hand, if this happens in the listener, PTRF passes the same frame several times to the application, causing the failure of the system.	Yes
Eliminating all replicas of a frame upon reception	Temporary faults may affect the identification of replicas upon reception in such a way that all the replicas of a specific frame are eliminated. If this happens the intended listeners do not receive the frame and, thus, they cannot carry out their operation properly.	Yes

**Table 4:** Reliability of TSN, approach A and approach B in the case of reference.

TSN	Approach A	Approach B
0.858467899141365	0.999958504043796	0.999943599679439

## Data Availability

Álvarez, Inés (2021), “Reliability Sensitivity Analyses”, Mendeley Data, V1, doi:10.17632/jnsgkc23xs.1.

## Abbreviations

The following abbreviations are used in this manuscript:

BER	Bit Error Rate
CRC	Cyclic Redundancy Check
COTS	Commercial Off The Shelf
CQF	Cyclic Queuing and Forwarding
DTMC	Discrete-Time Markov chains
FT	Fault Tolerance
FRER	Frame Replication and Elimination for Reliability
PTRF	Proactive Transmission of Replicated Frames
RT	Real-Time
TAS	Time-Aware Shaper
TG	Task Group
TSN	Time-Sensitive Networking
TT	Time-Triggered
